# An Optimised Step-by-Step Protocol for Measuring Relative Telomere Length

**DOI:** 10.3390/mps3020027

**Published:** 2020-04-03

**Authors:** Mugdha V. Joglekar, Sarang N. Satoor, Wilson K.M. Wong, Feifei Cheng, Ronald C.W. Ma, Anandwardhan A. Hardikar

**Affiliations:** 1 Diabetes and Islet biology, NHMRC Clinical Trials Centre, Faculty of Medicine and Health, University of Sydney, Camperdown, NSW 2150, Australia; sarangsatoor@gmail.com (S.N.S.); wilson.wong@ctc.usyd.edu.au (W.K.M.W.); 2 DNA Sequencing Laboratory, National Centre for Cell Science, NCMR Campus, Sai Trinity Complex, Pashan, Pune 411 021, India; 3 Department of Medicine & Therapeutics and Li Ka Shing Institute of Health Sciences, Chinese University of Hong Kong, Prince of Wales Hospital, Hong Kong, China; CHENG-Feifei@link.cuhk.edu.hk (F.C.);

**Keywords:** telomeres, real-time PCR, relative telomere length, cells, DNA

## Abstract

Telomeres represent the nucleotide repeat sequences at the ends of chromosomes and are essential for chromosome stability. They can shorten at each round of DNA replication mainly because of incomplete DNA synthesis of the lagging strand. Reduced relative telomere length is associated with aging and a range of disease states. Different methods such as terminal restriction fragment analysis, real-time quantitative PCR (qPCR) and fluorescence in situ hybridization are available to measure telomere length; however, the qPCR-based method is commonly used for large population-based studies. There are multiple variations across qPCR-based methods, including the choice of the single-copy gene, primer sequences, reagents, and data analysis methods in the different reported studies so far. Here, we provide a detailed step-by-step protocol that we have optimized and successfully tested in the hands of other users. This protocol will help researchers interested in measuring relative telomere lengths in cells or across larger clinical cohort/study samples to determine associations of telomere length with health and disease.

## 1. Introduction

Telomeres are part of the non-coding genome with distinct structure and function. They are short, repeat DNA sequences of 5’-TTAGGG-3’ at the ends of each chromosome in most eukaryotic cells [1,2,3]. The main function of telomeres is to protect the chromosomal ends from damage, which they achieve by forming a telomere loop (t-loop) around a six-protein complex called “shelterin” [4]. At each round of DNA replication, part of telomeres is successively lost, resulting in telomere shortening [5]. Reduced telomere length is associated with cellular aging [6], genetic diseases [7,8], cancers, and chronic diseases [9,10]. Measuring telomere length is, therefore, considered as an attractive biomarker for potential risk stratification in various diseases. 

Different methods are available to measure telomere length, each with its advantages and disadvantages. Main methodologies include terminal restriction fragment analysis (sometimes also referred as telomere restriction fragment analysis) followed by Southern blotting, real-time quantitative PCR (qPCR), quantitative Fluorescence In Situ Hybridization (Q-FISH), Single Telomere Length Analysis (STELA), and Telomere Shortest Length Assay (TeSLA) [11,12]. Quantitative PCR (qPCR)-based relative telomere length measurement is a high throughput method that requires small starting material, less time, simple procedures, and, therefore, ideal to quantify hundreds to thousands of samples from an epidemiological study. In 2002, Cawthon designed primers that could bind to GC-rich regions [13] and revolutionized telomere biology by making this qPCR method available for larger population-based clinical studies. 

In Cawthon’s method, telomere length (T) is typically compared to that of a single copy gene (S) by performing two different qPCR reactions, one using telomere primers and the other with single-copy gene primers. Since telomere is a repeat sequence of (TTAGGG)n, whereas single-copy gene is a unique single sequence of that gene within the genome, the results of qPCR yield the average relative telomere length (usually presented as T/S ratio). This method has its own limitations: it cannot measure absolute telomere length and the telomere length of the individual chromosome. Another drawback is variability in measurements/results that arise due to inconsistencies of protocols, reagents, and data analysis between different labs. Furthermore, the single-copy gene 36B4/RPLP0 (ribosomal protein lateral stalk subunit P0) in the Cawthon protocol has been used in different studies so far but is now known to have multiple processed pseudogenes, making it unsuitable as a single copy gene (RefSeq Jul 2008, [14]). 

High-quality DNA, telomere, and single-copy gene primers, master-mix, and a well-calibrated PCR instrument, are the basic essential factors to perform a successful qPCR-based relative telomere length analysis. Multiple studies have suggested that variations in the above factors lead to differences in data [15]. DNA extraction methods [16,17,18], DNA storage concentration and temperature [19,20], different commercially available master-mixes [21], PCR conditions, single-copy genes and instruments [14,22] are shown to alter telomere PCR readings. Another critical factor is data analysis and the use of reference/control samples for comparison. There are no standard guidelines on using the reference/control sample or calculating the T/S ratio, though 2^−ΔΔCt^ is a very common way of reporting relative telomere length, as suggested in the original Cawthon paper. 

Overall, the qPCR technique is the most commonly used high throughput method for telomere length measurement; however, it is reported to have inconsistencies between laboratories and between association studies on clinical cohorts. As discussed above, multiple variables play an important role in deciding the reproducibility and reliability of this technique. Even though each of the above variables is optimized by different groups to yield reproducible results, it may be overwhelming for a new user to start with such diverse information. We, therefore, provide here a step-by-step protocol including details of primers, reagents, PCR instruments, and data analysis steps. This protocol forms the most recent working qPCR-based method, which, although based on the original Cawthon protocol, presents an easy step-wise workflow. Our protocol has been tested by users with varied expertise in PCR techniques, in different laboratories as well as on two different platforms to yield significantly lower inter- and intra-assay coefficient of variations (CVs).

## 2. Experimental Design

For the demonstration of this protocol, we obtained around 10 mL of blood in a standard Vacutainer® EDTA tube from healthy volunteers (N = 3) after informed consent (HREC/13/RPAH/83). In a separate replication/validation study, 10 mL of blood was collected in a standard Vacutainer® EDTA tube from ethnically diverse (N = 21) individuals. All samples were obtained from individuals with a mean age of 48.8 ± 13.9 years (57% males) and were either Asian or Caucasian. Samples were mixed thoroughly by inversion and stored at −80 °C as 1 mL aliquots. A single aliquot of 1 mL blood was thawed on ice and used for DNA isolation and quantification, as described below. We understand that DNA isolation is a standard workflow in most laboratories, and several have their own preferences for reagents and commercially available kits. Any of these protocols yielding high quality, undegraded DNA could be used to obtain DNA required for telomere length measurement. Since the DNA isolation method is a known variable in telomere qPCR data, it is important to use the same DNA isolation method across all study samples for valid comparisons. We trialed three different kits for DNA isolation on a set of around 12 samples by three users (Figure S1). We observed that kit 3 yielded mostly intact DNA (single, intense band without smear), and results were consistent across three users. We, therefore, recommend using kit 3 (QIAamp™ Blood Midi kit from Qiagen^®^) with modifications detailed below. Each sample was diluted to achieve three concentrations (100 ng/μL, 25 ng/μL, 6.25 ng/ μL). The present protocol describes the results using these three samples. However, the steps are written for assessing relative telomere length in any epidemiological study. The timing is provided for measuring 30 samples at a time and on a single instrument.

In clinical studies, it is recommended that data related to age, gender, and ethnicity are recorded. In our protocol, we use 30 samples per plate in triplicate along with water/no template control (NTC) and a reference/control DNA (blood sample from a non-study sample/cell line DNA). Sample locations are kept identical when performing telomere and single-copy gene PCR. For longitudinal studies, samples from the same individual at different time points are kept on the same plate. Reference/control DNA (hereafter referred to as Control DNA) is used to calculate intra- and inter-assay CVs, while NTC is mainly used to understand PCR contamination. 

### 2.1. Materials

#### 2.1.1. DNA Isolation

- QIAamp™ Blood Midi kit (Qiagen^®^, Hilden, Germany; Cat. no.: 51185) or any other preferred/tested kit. The Qiagen^®^ kit contains QIAamp Midi Spin Columns, Collection Tubes (15 ml), Buffer AL, Buffer AW1 (concentrate), Buffer AW2 (concentrate), Buffer AE and QIAGEN^®^ Protease.
- Molecular biology grade ethanol (100% pure) (Sigma-Aldrich, St. Louis, MO, USA; Cat. no.: E7023)

#### 2.1.2. Telomere and Single Copy Gene Quantitation by qPCR

- Fast SYBR Green master-mix (ThermoFisher Scientific, Waltham, MA, USA; Cat. no.: 4385617)
- A fresh aliquot of nuclease-free water (Qiagen, Hilden, Germany; Cat. no.: 129117)
- Primers for telomere as detailed in [23] (Integrated DNA Technologies, Coralville, IA, USA; Cat. no.: CUSTOM OLIGOS)
  Telomere (A): CGGTTTGTTTGGGTTTGGGTTTGGGTTTGGGTTTGGGTT
  Telomere (B): GGCTTGCCTTACCCTTACCCTTACCCTTACCCTTACCCT
- Primers for human β-globin as detailed in [24] (Integrated DNA Technologies, Coralville, IA, USA; Cat. no.: CUSTOM OLIGOS). The human β-globin gene is used as single-copy gene in our protocol.
  hbg1: GCTTCTGACACAACTGTGTTCACTAGC
  hbg2: CACCAACTTCATCCACGTTCACC

#### 2.1.3. Consumables

- 1.7 mL Eppendorf tubes (Axygen Scientific, Union City, SF, USA; Cat. no.: MCT-175-C)
- P10, P20, P200 and P1000 Filtered pipette tips (Axygen Scientific, Union City, SF, USA; Cat. no.: TF-300-L-R-S, TF-100-L-R-S, TF-200-L-R-S, and TF-1000-L-R-S, respectively)
- 15 mL conical tubes (Sigma-Aldrich, St. Louis, MO, USA; Cat. no.: CLS430791)
- MicroAmp™ Fast Optical 96-well PCR plates (0.1 mL volume of each well) (ThermoFisher Scientific, Waltham, MA, USA; Cat. no.: 4366932)
- MicroAmp™ Optical Adhesive Film for the PCR plates (ThermoFisher Scientific, Waltham, MA, USA; Cat. no.: 4311971)

### 2.2. Equipment

- Water bath that can heat up to 70 °C (Polysciences, Philadelphia, PA, USA; Cat. no.: WBE20A12E).
- ViiA7 qPCR machine with fast 96-well blocks (ThermoFisher Scientific, Waltham, MA, USA; Cat. no.: 4453535)
- Nanodrop-2000 (ThermoFisher Scientific, Waltham, MA, USA; Cat. no.: ND-2000)
- Vortex mixer (Heidolph, Schwabach, Germany; Cat. no.: 541-10000-00)
- Refrigerated centrifuge with plate adapters (Eppendorf, Hamburg, Germany; Cat. no.: 5811000088 and 5810725003, respectively)
- Sprout^®^ Mini Centrifuge (Heathrow Scientific, Vernon Hills, IL, USA; Cat. no.: HS120301)
- Well calibrated micropipettes (as indicated) (ThermoFisher Scientific, Waltham, MA, USA; Cat. no.: 4700880)

## 3. Procedure

### 3.1. DNA Isolation. Time for Completion of 30 Samples: 02:30 h

- Resuspend the blood sample (total 1 mL) thoroughly by inversion and transfer the 900 μL of blood into a fresh 15 mL conical tube.
- Follow the steps as detailed in the QIAamp™ Blood Midi kit handbook. We obtain consistent results with a minimum variation using this kit and recommend the following minor modifications.
- Molecular biology grade ethanol 100%, pure and fresh, is recommended than 96–100% (Qiagen^®^ protocol).
- Use nuclease-free cut filter tips rather than regular filter tips. This allows for easier workflow when dealing with high viscosity blood samples.
- Handling of 30 samples at a time can be a bit tricky. This aspect is described in the higher throughput portion of the kit protocol. We recommend placing all 30 isolation tubes in a single rack and clamping it to another rack with elastic bands. We suggest mixing samples by using a flat head Vortex mixer at the lysis step. The following steps (ethanol addition) could use inversion or vortexing.
- We observe better yield and quality when using a water bath incubator for 70 °C incubation and extending the incubation time to 15 min.

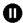
**PAUSE STEP**: If using the QIAamp™ kit, elute DNA in buffer AE and aliquot for long-term storage into multiple nuclease-free tubes to be stored at −80 °C. 

### 3.2. DNA Quantitation and Dilution. Time for Completion: 01:00 h for 30 Samples

- Quantify DNA samples and Control DNA on Nanodrop. The 260/280 ratio for isolated DNA using the modified QIAamp^TM^; Blood Midi kit protocol is ≥1.8. DNA quality can be assessed using agarose gel-based separation confirming the absence of any degraded DNA in the form of a smear on the gel (Figure S1).
- Dilute each DNA sample to a required concentration. Here we used three different concentrations during the optimization stage. With the actual experiments (with clinical samples), we use a concentration of 25 ng/μL in a total volume of 8 μL (i.e., 200 ng of total DNA in 8 μL of total volume). Dilutions are performed with the addition of nuclease-free water.
- Spin and store undiluted DNA samples at −80 °C for long-term storage.

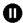
**PAUSE STEP**: Diluted DNA can be stored at −80 °C for up to one week.

### 3.3. Monoplex Real-time PCR. Time for Completion: 04:00 h for 30 samples and Two Genes 

#### 3.3.1. Preparation of the Master-Mix

- Create the master-mix as per the volumes of each reagent indicated per reaction (below, Table 1).
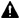
**CRITICAL STEP:** It is important to add 5% extra to account for pipetting errors. This ensures that you have enough master-mix for the desired number of reactions that are planned. See an example in Table 1 below.
- Reconstitute primers with nuclease-free water to a stock concentration of 5 mM.
- Dilute the telomere A primer stock serially to 100 μM and then to 1μM (working concentration) using nuclease-free water.
- Dilute the telomere B primer stock serially to 100 μM and then to 3μM (working concentration) using nuclease-free water.
- Dilute the hbg1 primer stock serially to 100 μM and then to 3μM (working concentration) using nuclease-free water.
- Dilute the hbg2 primer stock serially to 100 μM and then to 7μM (working concentration) using nuclease-free water.
- Vortex and spin the SYBR green master-mix and primers before addition.
- Prepare two master-mixes, one for telomere PCR that contains telomere A and telomere B primers; whereas another one for single-copy gene PCR contains hbg1 and hbg2 primers.

#### 3.3.2. Plate Setup

**Figure 1:**
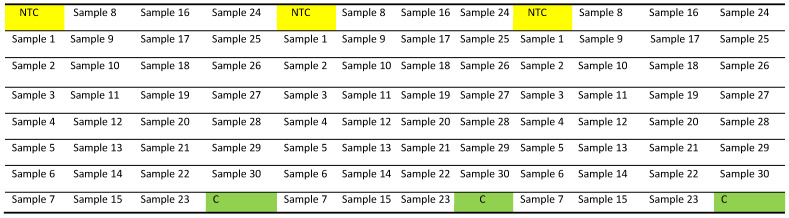
PCR plate map. A template of 96-well plate for telomere PCR that accommodates triplicates of no template control (NTC), 30 samples, and Control DNA (C) in different positions as indicated. Exact same PCR is setup for single-copy gene (human β-globin) with identical well positions for NTC, samples, and Control DNA.

- Briefly vortex and centrifuge the master-mix.
- Pipette 9 μL of the above mixture (either for telomere or human β-globin) into each well of a Fast Optical 96-Well Reaction Plate (0.1 mL), then pipette 1 μL of Nuclease-free water (for no template control/NTC), DNA sample or Control DNA (C), in accordance with the 96-well plate map shown below (Figure 1). We recommend having each sample in triplicate, including the NTC and Control DNA.

- There will be two different plates for each sample. One plate containing telomere primers to measure relative telomere abundance and other plates containing human β-globin primers to measure relative single-copy gene abundance.
- Apply an optical adhesive film over the top of the plate, ensuring there is a complete seal and minimizing the generation of any aerosols at this point.
- Centrifuge the plate at 1800 g (3000 rpm) for 5 min at 4 °C. Make sure that there are no air bubbles in these wells. If present, gently flicks to remove bubbles and centrifuge again at 3000 rpm for a further 2 min.

#### 3.3.3. Real-Time PCR

**Figure 2:**
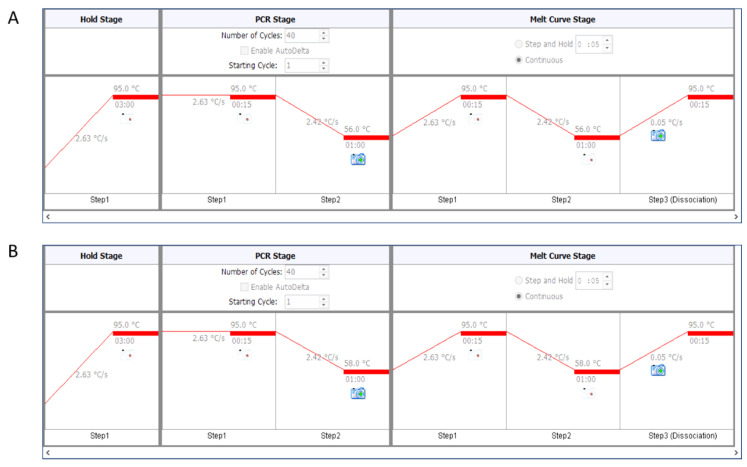
Details of the PCR setup. Detailed information of the PCR setup on the ViiA7 instrument with telomere primers (**A**; annealing at 56 °C) and human β-globin primers (**B**; annealing at 58 °C) is provided. Cycling temperatures with time, number of cycles, as well as ramping rates are clearly indicated here. A melt curve analysis is added at the end of 40 cycles of PCR to ensure the absence of primer-dimers.

- Insert the plate into the ViiA™ 7 Real-Time PCR System (CONFIRM CORRECT ORIENTATION), load up the Quantstudio Real-time PCR software on the computer and click Experiment Setup.
- Select ViiA7 instrument, Fast 96 Well (0.1 mL) block, Standard Curve, SYBR Green reagents, and FAST options from the setup.
- Enter the target names and sample names and assign correct labels to the wells. Enter reaction volume of 10 μL.
- For telomere PCR, use 56 °C as annealing temperature (Figure 2A).
- For single-copy genes (here human β-globin), use 58 °C as annealing temperature (Figure 2B).
- To start the reaction, save the run and then click START RUN.

### 3.4. Data Analysis. Time for Completion: 01:00 h

**Figure 3:**
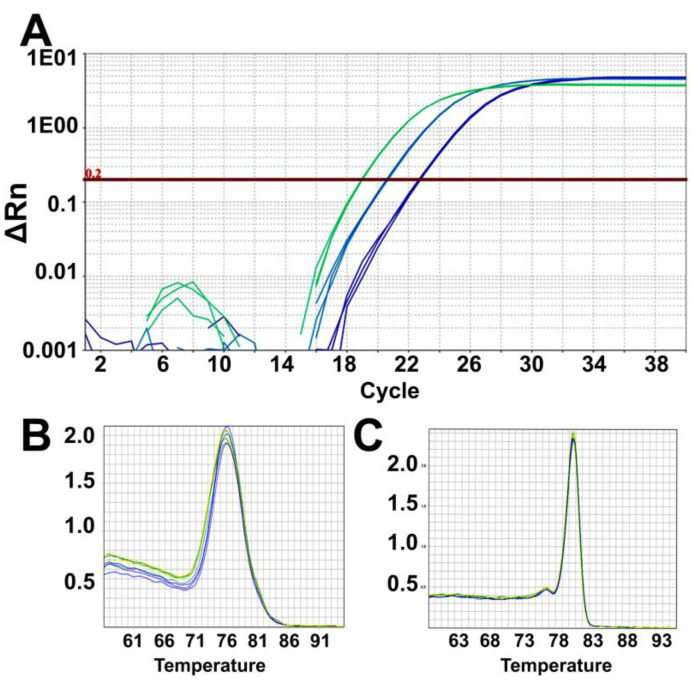
Expected PCR results. (**A**) representative amplification plot for three DNA concentrations performed in triplicates (100 ng/μL, green curves; 25 ng/μL, cyan curves and 6.25 ng/μL, blue curves). All replicates have almost identical cycle threshold value, and for each four-fold dilution, the cycle threshold values increase by two cycles. Representative images of the melt curve for telomere PCR products (**B**) and human β-globin PCR products (**C**).

- Check that the amplification curves are exponential, and the threshold is set in the linear (0.2) range of the curves (Figure 3A). Melt curves show a single peak for telomere (Figure 3B) and human β-globin (Figure 3C) reactions, indicating the presence of a single amplicon.
- Analyze the data using the same threshold for all samples in the Quantstudio real-time PCR software and then export the results as an excel sheet. Results are analyzed and plotted using Excel and GraphPad Prism.
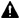
**CRITICAL STEP:** It is important to keep the same threshold (such as 0.2) across all the plates from where data is to be analyzed.
- Organize the results as triplicates cycle threshold values (Ct) of telomere and human β-globin genes for each sample, NTC, and Control DNA (Supplementary Table S1).
- Calculate average Ct and standard deviation of each gene for each sample, NTC, and Control DNA.
- Calculate ΔCt for each sample as (Average Ct of hbg)-(Average Ct of telomere). Since telomeres are present in multiple copies in a cell, as against single-copy genes, ΔCt value is positive.
- Similarly, calculate Control DNA ΔCt values.
- Calculate relative telomere length as ∆∆Ct = (Sample Average hbg Ct-Sample Average Telomere Ct)–(Control DNA Average hbg Ct-Control DNA Average Telomere Ct). This value can be represented as the relative telomere length (RTL).
- Calculate the intra-assay CV between the replicate values of Control DNA in the same plate and inter-assay CV between the replicate values of Control DNA from at least three different plates.

## 4. Results and Discussion

**Figure 4:**
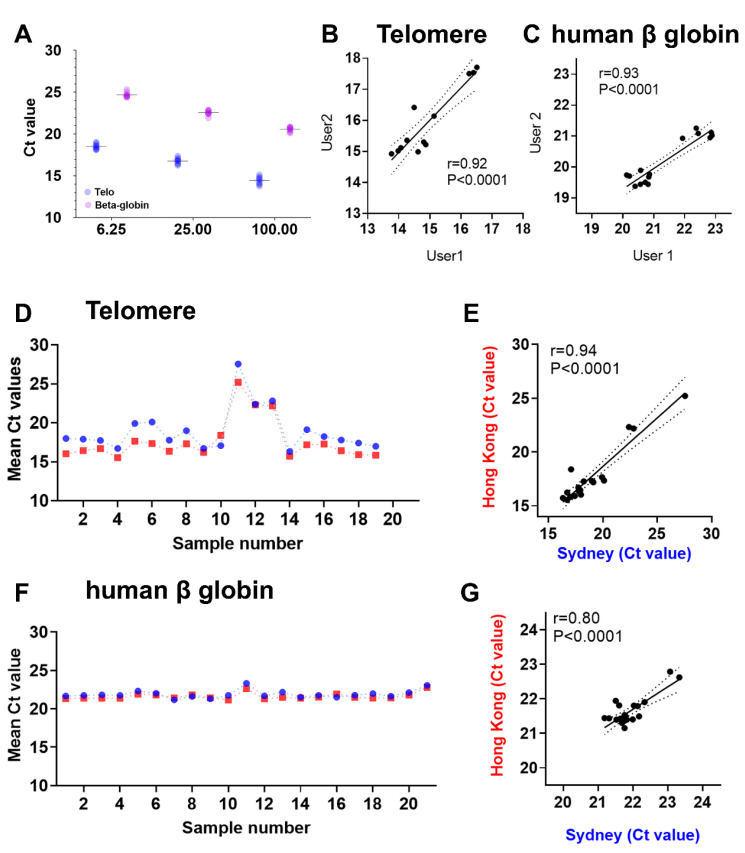
Reproducibility of data: the actual cycle threshold (Ct) values for the three different dilutions (100 ng/μL, 25 ng/μL, 6.25 ng/μL) used in the qPCR reactions for telomere and human β-globin expression from three technical repeats and three biological samples (**A**). PCR data from the same set of samples were obtained by two different users from the same lab, and the correlation between their results are plotted for telomere (**B**) and the human β-globin gene (**C**). Reproducibility of the method across two different labs (Sydney; Blue circles and Hong Kong; Red squares) is presented as mean Ct values of the triplicates obtained after telomere qPCR (**D**) and human β-globin qPCR (**F**). Correlation plots for telomere qPCR data (**E**) and human β-globin qPCR data (**G**) performed in each lab on the same samples. B,C,E, and G show Pearson (r) correlation coefficient and p-values.

We typically obtain the dilution results for telomeres and single-copy genes, as shown in Figure 3 and Figure 4. Three samples at three different dilutions (100 ng/μL, 25 ng/μL, 6.25 ng/μL) were used in the PCR reactions, as described above to obtain Ct values for telomere and human β-globin expression. We obtain close to two Ct value increases for each four-fold dilution in all the three samples shown here (Figure 3A and Figure 4A). The Pearson correlation values are very high for both the telomere and human β-globin genes (r^2^ = 0.93 and r^2^ = 0.92, respectively: Figure 4A). PCR efficiencies are observed to be 97.12% with confidence intervals of 85.55% to 112.17% for telomeres and 95.22% with confidence intervals of 86.99% to 105.17% for the human β-globin gene. It is essential to calculate and ensure equal PCR efficiencies prior to assessing relative telomere lengths using ∆∆Ct method as described above. 

With our protocol, we observe very low CVs with intra-assay CV <2%, inter-assay CV <4%, similar PCR efficiencies for telomeres and single-copy genes in an acceptable range of 90–100%, linear dynamic range of amplification between 7–34 cycles, and almost undetectable signal in NTC. Our QC steps include repeating all the samples for both telomere and human β-globin if any of the PCR replicates, for that sample is beyond 2% of error margin.

As shown in Supplementary Table S1, we first calculated average and standard deviation of cycle threshold values for three technical repeats of each sample on telomere and human β-globin gene PCR. The average values are used to calculate ΔCt for each sample and ΔΔCt representing relative telomere length as detailed in Section 3.4 above. Supplementary Table S1 provides details of results and formulas for ΔΔCt calculations of the three samples tested here. A higher ∆∆Ct value indicates a longer telomere length. We recommend using a control sample (non-study sample/cell line DNA/reference DNA of known telomere length) for ΔΔCt calculations in actual epidemiological/clinical study data reporting. 

The reproducibility of the data has been a concern in qPCR-based workflows for telomere length measurements [15]. Our protocol has been tested by multiple users from different laboratories. We observe high reproducibility in qPCR data for telomere (Figure 4B) and human β-globin gene (Figure 4C) with correlation coefficients >0.9 in the hands of two different users from the same lab. Similarly, we tested our protocol on the same set of samples in two different labs (Sydney and Hong Kong). The cycle threshold values for both telomere and human β-globin were highly overlapping (Figure 4D,F), yielding a correlation coefficient of 0.94 for telomere (Figure 4E) and 0.8 for human β-globin (Figure 4G).

We trialed different DNA isolation kits (Figure S1) and then optimized single-copy gene primers using DNA isolation methodology that delivered high-quality DNA. Multiple gene candidates are reported to be used as single-copy genes in the literature. We started with 36B4, as first reported in the Cawthon paper [13]. However, we observed significant variation between different batches of 36B4 primers as well as in different aliquots of the same stock of primers. This prompted us to trial other single-copy genes, including Glyceraldehyde 3-phosphate dehydrogenase (GAPDH), as well as the human β-globin gene. We found the human β-globin gene to yield consistent results irrespective of the supplier, batch/lot, and freeze-thaw cycles, and therefore propose to use this as a single copy gene in the current optimized protocol. It is important to select a single copy gene that provides reproducible output across different age groups, ethnicities, and tissue types. 

Another note of caution is the reaction volumes and the detection platform. We have observed that our protocol works best on the ViiA7 machine in a 96-well plate with 10 μL total reaction volume. We have observed that it can be performed in a 384 well plate on an OpenArray platform. We believe that our protocol is very robust and can be adapted to other PCR machines. We, however, recommend optimization with at least 3–4 control samples at four different dilutions before changing the PCR reaction volumes and platforms to ensure assay reproducibility. It will be ideal for verifying the change in telomere lengths identified using this protocol with other complementary [15] or alternate techniques such as those involving the assessment of intracellular telomerase activity.

Automation platforms can be optimized to further increase the throughput and precision during telomere length measurement by qPCR. We have trialed the QIAcube HT robotic platform to isolate DNA from 96 samples at a time or aliquot master-mix and DNA with higher precision into PCR plates. Other robotic, automated platforms such as epMotion^®^, Biomek could be used along with specifically customized workflows to increase the efficiency and reproducibility of qPCR data for telomere and single-copy genes. Additionally, the use of duplexing with TaqMan primer-probes could further reduce the time on performing these assays in the future.

A common practice in telomere length analyses has been the use of one (or a pool) of the clinical study DNA samples as a control sample. This practice limits the replication of such data in other laboratories that may not have access to the same patient pool/control samples. As concluded by Martin-Ruiz [22], standard control material that is available to all labs engaged in telomere measurement is necessary. We think that using a non-study sample such as a well-defined commercially available reference DNA or cell line [25] is a more appropriate control for data normalization step, especially when comparing study results in different cohorts across different laboratories worldwide. 

Similar to the use of various candidates as single-copy genes and the lack of a consensus around using a control sample, there are differences in calculating the relative telomere lengths (See Supplementary Table S2). Some studies report ΔCt or −ΔCt or 2^−ΔCt^ values presenting differences between single-copy gene and telomere Ct values whilst others report ΔΔCt or −ΔΔCt or 2^−ΔΔCt^ values where differences between single-copy gene and telomere Ct values for a particular sample are compared to that of a control sample. The T/S ratio, presented as the Ct-value of (T) relative to (S) is also calculated as (Average Ct of telomere)-(Average Ct of hbg). Here the ΔCt value will be negative and hence demonstrated by some laboratories as −ΔCt. All these methodologies are valid as long as the same data analyses are applied while comparing different samples/patient datasets within a study. However, cross-study comparisons cannot be made between the relative telomere length measurements computed using different methods and DNA controls. Since the qPCR-based protocol described here provides a relative abundance of telomeres, care should be taken when reporting study results in terms of whether the calculations are providing telomere length relative to single-copy gene (T/S) or telomere length relative to a control sample (RTL).

Overall, qPCR-based methodology offers a simple, high-throughput, quick, and straight forward workflow for the measurement of relative telomere length with the potential to upscale using automation. Accurate reporting of the control sample, single-copy gene, and calculations is essential for all the studies performed using qPCR-based methods. This protocol demonstrates the usability of such a highly reproducible and robust qPCR-based workflow across users at different skill levels and laboratories that are geographically distinct. We believe that our detailed protocol will be helpful to researchers in designing, performing, and reporting their experiments as well as making informed decisions while selecting single-copy genes, control samples, PCR platforms, and methods for calculating their study results. 

## 5. Reagents Setup

- All plasticware should be nuclease-free. Never stick your hands into containers of nuclease-free plastic ware. Always pour out the required tubes.
- The use of appropriate PPE is a must.
- Equilibrate blood samples to room temperature (15–25 °C) before starting by placing a batch of 30 samples in a rack at room temperature.
- Follow the DNA isolation protocol provided by the manufacturer. If using the QIAamp™ kit, prepare buffers for DNA isolation as recommended in the QIAamp™ Blood Midi kit handbook and also setup the water bath at 70 °C before initiating DNA isolation.
- All reagents for PCR are stored at −20 °C. Allow samples, primers, and master-mix to thaw thoroughly on ice prior to commencing.
- Master-mix can be aliquoted into smaller volumes (500 μL) to avoid multiple freeze thaws.
- Reconstitute the primers and then dilute them serially to 100 μM first and then to the lower working concentration, as mentioned in the procedures above.
- Make enough volumes of the dilutions for multiple runs in advance. 100 μL of the working concentration primers are required to run one 96-well plate.
- Avoid freeze-thaw of primers and Control DNA samples.

## Figures and Tables

**Table 1:** Preparation for PCR reactions. Reagents and their volumes per PCR reaction are provided. We also indicate the volumes needed for 96 reactions that include a 5% extra amount to accommodate pipetting errors.

Reagent	Volume/Reaction (μL)	Volume for 96 Reactions (μL)
2X Fast SYBR^®^ Green Master Mix	5	504
Telomere A OR hbg1 primer (working concentration)	1	100.8
Telomere B OR hbg2 primer (working concentration)	1	100.8
Nuclease-free water	2	201.6
**TOTAL**	**9**	

## Supplementary Materials

The following are available online at https://www.mdpi.com/2409-9279/3/2/27/s1, Figure S1: Optimization of DNA isolation kits, Table S1: PCR data and relative telomere length calculations excel sheet, Table S2: Existing differences in qPCR-based telomere length measurement methods.
