# Supplementary material for: An Optimised Step-by-Step Protocol for Measuring Relative Telomere Length
Source: Methods Protoc. 2020 Apr 3;3(2):27. doi: 10.3390/mps3020027 (PMC7359711; doi:10.3390/mps3020027)
Supplement: Supplementary file 1 [file mps-03-00027-s001.zip › mps-741218-sp/mps-741218-sp.docx]

Protocol

An Optimised Step-by-Step Protocol for Measuring Relative Telomere Length

Mugdha V. Joglekar ^1,^ *, Sarang N. Satoor ^1,2^, Wilson K.M. Wong ^1^, Feifei Cheng ^3^, Ronald C.W. Ma ^3^ and Anandwardhan A. Hardikar ^1^ *

1. Diabetes and Islet biology, NHMRC Clinical Trials Centre, Faculty of Medicine and Health, University of Sydney, Camperdown, NSW 2150 Australia; [sarangsatoor@gmail.com](mailto:sarangsatoor@gmail.com) (S.N.S.); [wilson.wong@ctc.usyd.edu.au](mailto:wilson.wong@ctc.usyd.edu.au) (W.K.M.W.)
2. DNA Sequencing Laboratory, National Centre for Cell Science, NCMR Campus, Sai Trinity Complex, Pashan, Pune 411 021, India
3. Department of Medicine & Therapeutics and Li Ka Shing Institute of Health Sciences, Chinese University of Hong Kong, Prince of Wales Hospital, Hong Kong, China; [CHENG-Feifei@link.cuhk.edu.hk](mailto:CHENG-Feifei@link.cuhk.edu.hk)

***** Correspondence: [mugdha.joglekar@ctc.usyd.edu.au](mailto:mugdha.joglekar@ctc.usyd.edu.au) (M.V.J.); [anand.hardikar@ctc.usyd.edu.au](mailto:anand.hardikar@ctc.usyd.edu.au) (A.A.H.)

Tel.: +61 2 9562 5071 (A.A.H.); +61 2 9562 5084 (M.V.J.)

Received: 24 February 2020; Accepted: 31 March 2020; Published: date


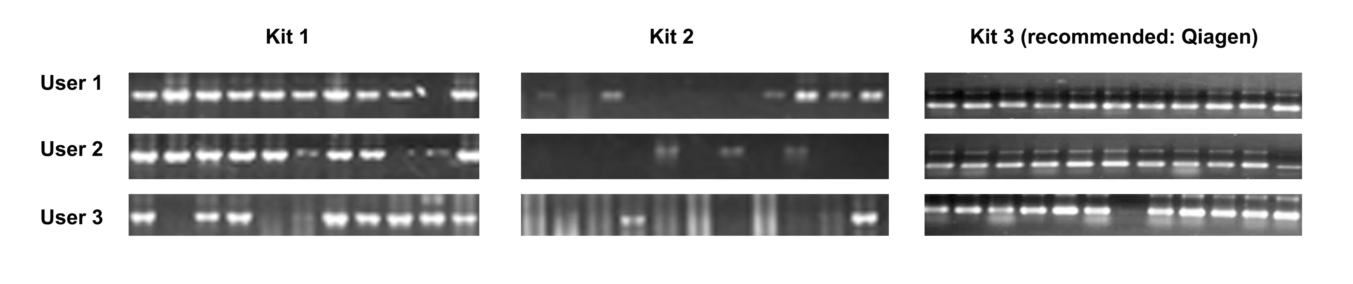


**Figure 1.** Optimization of DNA isolation kits. Images of agarose gels used to test the quality of DNA isolated from around 12 blood samples using three different kits (Kit 1, 2, 3) by three different users with different levels of expertise.
